# Microbe Profile: Paracoccus denitrificans - a versatile model

**DOI:** 10.1099/mic.0.001644

**Published:** 2026-01-29

**Authors:** Stephen Spiro, David J. Richardson

**Affiliations:** 1 Department of Biological Sciences, University of Texas at Dallas, Richardson, Texas 75080, USA; 2 School of Biological Sciences, University of East Anglia, Norwich, Norfolk, NR4 7TJ, UK

**Keywords:** denitrification, methylotrophy, oxidative phosphorylation, *Paracoccus denitrificans*

## Abstract

*Paracoccus denitrificans* is a metabolically versatile alphaproteobacterium first isolated in 1910 by Martinus Beijerinck. Similarities between the aerobic respiratory chain and membrane composition of *P. denitrificans* and those of eukaryotic mitochondria have stimulated the use of *P. denitrificans* as a model for oxidative phosphorylation. The organism has also been used extensively as a model for studies of denitrification, cytochrome c biogenesis, lithotrophy using thiosulphate as a source of energy, methylotrophy, and carbon metabolism more broadly. Through the application of structural biology and modern genome-based approaches, work on *P. denitrificans* continues to make significant contributions across multiple areas of microbiology.

## Graphical abstract

Electron transfer pathways of *Paracoccus denitrificans*. Enzymes and electron carriers are coloured according to location: orange (membrane bound) and blue (periplasmic). Electron transfer reactions are coloured black, blue and orange to indicate that they occur predominantly in the presence or absence of oxygen, or both, respectively. Note that pseudoazurin is expressed under anaerobic growth conditions and donates electrons to the nitrite, NO and N_2_O reductases. Abbreviations: ETF, electron transfer flavoprotein; NAR, membrane-bound nitrate reductase; NAP, periplasmic nitrate reductase; MDH, methanol dehydrogenase; MaDH, methylamine dehydrogenase; AMI, amicyanin; SOX, sulphur oxidation system; ASD, aldose sugar dehydrogenase; UQ, ubiquinone; UQH_2_, ubiquinol.

## Taxonomy

Domain *Bacteria*, phylum *Proteobacteria* (*Pseudomonodota*), class alpha subdivision, family *Paracoccaceae*, species *Paracoccus denitrificans*.

## Properties

*P. denitrificans* is a Gram-negative facultative anaerobe with non-motile coccoid cells typically around 1 µm in diameter. The optimal temperature for growth is in the range 30–36 °C. The organism is oxidase-positive, and energy is obtained from a wide array of organic substrates, including one-carbon compounds (methanol, methylamine, formaldehyde and formate). Chemolithotrophic growth is possible with hydrogen or reduced sulphur compounds as electron donors. Fermentative metabolism is absent; energy conservation under anaerobic growth conditions is by denitrification, the reduction of nitrate and nitrite to nitric oxide, nitrous oxide and dinitrogen, reactions that are coupled to the generation of proton motive force (PMF). Some strains are also able to couple heterotrophic nitrification (ammonia oxidation to nitrite) to aerobic denitrification, likely as a means of dissipating excess reductant.

## Genome

*P. denitrificans* strain Pd1222 has a genome that is 67% G+C and is organized into three replicons of 2.85, 1.73 and 0.65 Mbp. Strain ATCC 19637 has one chromosome (4.1 Mbp) and two plasmids (0.69 and 0.39 Mbp). Strain R1 has one chromosome of 4.05 Mbp and one plasmid of 0.69 Mbp. Of 28 other *Paracoccus* species listed in the KEGG database, 25 are annotated to have a single chromosome, the remaining three having two. Eighteen genomes have more than one plasmid (although numbers may be skewed in some cases by sequence gaps).

## Phylogeny

*P. denitrificans* (originally *Micrococcus denitrificans*) was isolated by Beijerinck from nitrate-reducing cultures. The genus *Paracoccus*, of which *P. denitrificans* was the type species, was first defined in 1969. Recent taxonomic proposals have seen the family *Rhodobacteraceae* renamed as *Paracoccaceae,* with *Paracoccus* being the type genus. The genus *Paracoccus* is the largest lineage in the family, currently having over 80 species isolated from diverse environments.

## Key features and discoveries

*P. denitrificans* is closely related to the mitochondrial ancestor and, for this reason, has long been used as a model for studies of oxidative phosphorylation. Structural information is available for NADH dehydrogenase (Complex I, [1]), the cytochrome *bc*_1_ complex (Complex III), cytochrome *c* oxidase (Complex IV), the intact F-ATP synthase [2], and for an NADH oxidase supercomplex containing Complexes I, III and IV in a 1 : 4 : 4 ratio. In addition to two isoforms of the *aa*_3_-type cytochrome *c* oxidase (which is similar to the mitochondrial Complex IV), oxygen can also be reduced by a high-affinity *cbb*_3_-type cytochrome *c* oxidase and by *ba*_3_ and *bd*-type quinol oxidases. Electrons released by the periplasmic oxidations of methanol, methylamine and reduced sulphur compounds are transferred (via small soluble electron carriers) to the *aa*_3_-type cytochrome *c* oxidase, with concomitant generation of PMF.

 In the absence of oxygen, PMF generation is coupled to the reduction of nitrate, nitrite, nitric oxide (NO) and nitrous oxide (N_2_O) with dinitrogen (N_2_) as the end product. Structures have been solved for oxidoreductases involved in this denitrification pathway, including the first structure of a periplasmic NO-generating cytochrome *cd*_1_ nitrite reductase [3]. Three distinct nitrate reductase systems are expressed: Nar, involved in anaerobic energy-conserving nitrate respiration; Nap, involved in aerobic energy-dissipating nitrate respiration; and Nas, which is required for nitrate assimilation.

In cultures transitioning from oxic to anoxic growth conditions, only a fraction of the population switches on expression of the complete denitrification pathway. This is proposed to be a bet-hedging strategy that protects against fluctuating oxygen availabilities [4]. Regulation of expression of denitrification genes is governed in part by FNR/CRP family members that respond to oxygen and NO, and there is evidence for gene regulation by small RNAs. Expression of the copper-containing N_2_O reductase is regulated by copper, and there is evidence that N_2_O also regulates gene expression by interacting with vitamin B12-containing riboswitches [5].

 One-carbon growth substrates are oxidized to carbon dioxide, which is then fixed into biomass by the Calvin–Benson cycle. *P. denitrificans* is the prototypical ‘autotrophic methylotroph’, although other members of the genus can assimilate C1 compounds via the serine cycle. *P. denitrificans* was among the first methylotrophs from which genes involved in C1 metabolism were cloned and characterized [6]. This included the first sequence of an *xoxF* gene, later shown in other organisms to encode a lanthanide-dependent methanol dehydrogenase (that substitutes for the alternative calcium-dependent enzyme under appropriate growth conditions). Complex regulatory networks involving multiple two-component regulatory systems coordinate expression of the genes encoding enzymes involved in methylotrophic metabolism.

 Work on *P. denitrificans* has made other significant contributions to our broader understanding of carbon and energy metabolism. One example is the detailed description of the β-hydroxyaspartate cycle [7] that is required for growth on glycolate, glyoxylate and ethylene glycol and was first proposed in the early 1960s by Hans Kornberg and J. Gareth Morris.

 Extensive genetic, biochemical and physiological characterization, together with a genome-wide metabolic model [8] and modern high-throughput approaches, means that *P. denitrificans* continues to provide valuable insights into diverse metabolic and regulatory processes.

## Open questions

- What are the roles of small RNAs and riboswitches in the regulation of the expression of denitrification genes?
- What is the ecophysiological role of phenotypic heterogeneity (‘bet hedging’)?
- Is there an active lanthanide-dependent methanol dehydrogenase, and, if so, what is the mechanism of reciprocal regulation of the two methanol dehydrogenases (the ‘lanthanide switch’)?

## Abbreviations

CRP: cAMP receptor protein
FNR: Regulator of fumarate and nitrate reduction
KEGG: Kyoto Encyclopedia of Genes and Genomes
Mbp: Megabase pairs
NADH: Nicotinamide adenine dinucleotide, reduced form
PMF: proton motive force
